# Observational research on fundamental nursing care: Enough already!

**DOI:** 10.1111/jocn.15203

**Published:** 2020-02-14

**Authors:** David A. Richards

**Affiliations:** ^1^ College of Medicine and Health University of Exeter Exeter UK

It is now ten years since Kitson and colleagues (Kitson, Conroy, Wengstrom, Profetto‐McGrath, & Robertson‐Malt, 2010) were wrestling with the notion of fundamental nursing care, a process that culminated seven years later in a consensus‐derived standardised definition for fundamental care and the discrete elements that constitute it (Feo et al., 2018). In 2018, as momentum gathered, the Journal of Clinical Nursing published its first special edition focussing on this most essential nursing care activity. Containing 28 articles by authors representing 16 countries from five of the seven continents (nothing from Africa sadly), Kitson's group and others can certainly be said to have placed fundamental nursing care firmly on the global map.

As such, they can be said to have successfully actioned one of the five propositions articulated in the “ILC Aalborg Statement” (Kitson et al., 2019): to “talk” fundamental care. Of the other four propositions, three are difficult to assess—“valuing,” “doing” and “owning”—being properties of the systems, groups and individuals that provide nursing care to individuals and families in need of it. The fifth proposition, that of “researching” fundamental care is easier to scrutinise, and it is that to which the rest of this editorial will turn its attention.

The ILC Aalborg statement proposes that “fundamental care must undergo systematic and high‐quality investigations to generate the evidence needed to inform care practices and shape health systems and educational curricula” (Kitson et al., 2019). In an era of evidence‐based practice (Sackett, Rosenberg, Gray, Haynes, & Richardson, 1996), such sentiments fit squarely within the current scientific paradigm driving improvements in health care. Few would argue against the notion that science, clinical expertise and patient preferences should define our practice—medical or nursing. However, as in all fields of human endeavour, nothing is quite as simple as it might appear.

In 2009, coincidently at the time that the concept of fundamental care was (re)emerging, Chalmers and Glasziou (Chalmers & Glasziou, 2009) published their astonishing analysis of medical research, arguing that 85% of all published research was “waste.” Their empirically based reasoning was that a combination of inappropriate focus, poor methods and biased and incomplete reporting reduced the value of most studies to the point where only 15% could be classed as useful for clinicians. Essentially, they stated that billions of dollars/pounds/euros etc. are being flushed down the drain annually in useless medical research.

To accept the arguments advanced by Chalmers and Glasziou, one has to believe that healthcare research should: firstly provide data on causation; that is, that actions performed in the process of delivering health care reliably produce a positive effect on the target symptoms, emotions or experiences of those receiving that health care; and secondly, should use methods that are as rigorous and controlled for bias as possible. The first point requires us to experiment, since without experimentation, we can generally only claim correlation not causation. The second point should be applied to any research undertaken at all, whatever method is selected.

In both editorials within the 2018 special edition (Jackson & Kozlowska, 2018; Kitson, 2018), the editors acknowledged that in terms of the evidence base for fundamental nursing care, practising nurses are not well served by nurse scientists. Jackson & Kozlowska noted: “there are many key areas of nursing practice that suffer because we have failed to provide rigorous evidence to support it. Fundamental care is such an area…” (Jackson & Kozlowska, 2018) and Kitson asked, “Is nursing's work really the last evidence‐free zone in health care? And, if so, why is this the case and what can we do to turn things around?” (Kitson, 2018). They drew their pessimism partly from a seminal systematic review (Richards, Hilli, Pentecost, Goodwin, & Frost, 2018) published in that first special edition that examined the quality of 149 experimental studies undertaken in four areas of fundamental care (i.e. studies which met the first experimental research criteria referred to above) only to find that a mere 13 (9%) were as free of potential bias as possible (largely failing the second quality criteria).

One way to reflect on these findings would be to assume we are in fact half way to meeting the calls from these editors for more and better research. We only have to improve the quality of the experimental studies currently being undertaken and out of this will emerge a vigorous and robust evidence base for fundamental nursing care. Unfortunately, one only has to examine the articles published in both special editions to be rapidly disabused of this notion. In the 2018 special edition, there was just one experimental study out of 28 articles, the remainder of which were six discussion papers, two editorials, two systematic reviews, two scoping reviews, one nonsystematic review, one Delphi study, one quality improvement report and twelve observational studies (ten of which were qualitative studies and two analysed quantitative data). In this (2020) special edition, we have a similar picture, with again only one experimental study out of the 19 papers, with the remainder being either reviews (four in total) or predominantly qualitative observational studies (ten papers).

Of course, these two special editions can only be considered as a very biased sample of the literature. Nonetheless, these observations chime with what we know in general about the scientific behaviour of nursing academics. Two reviews of all the articles published in the top 20 nursing journals by impact factor in 2010 and 2013, respectively, by European nurse scientists (Richards, Coulthard, & Borglin, 2014; Richards, Hanssen, & Borglin, 2018) show that these scientists predominantly publish observational research, equally split between qualitative and quantitative methods. Observational methods outweighed experimental research by a factor of 7 to 1 in 2010 and 4 to 1 in 2013. Of the 10%–20% of published research that was experimental, only half adopted the most rigorous designs involving both randomisation and control comparison groups. It would seem, therefore, that much of nursing research in general and indeed research into fundamental nursing care practices falls into Chalmers and Glaszious’ definition of waste (Chalmers & Glasziou, 2009).

This is not to universally denigrate the importance of “discovery” research, which can be used to flush out important phenomena and help us understand the needs of patients and/or practitioners. However, within an evidence‐based paradigm aimed at improving the experience and effectiveness of health care delivered by nurses, at some point, discovery must lead to intervention development, testing, evaluation and implementation. There is little evidence from papers submitted to and published in these two special editions that the process of transferring insights from observational data into testable, and crucially, *useful* fundamental nursing care interventions is currently underway.

Some will undoubtedly argue that nursing is just too complex an action to permit it being sufficiently confined for examination in experimental studies. However, nursing is by no means the only field to have faced this dilemma. For more than 20 years, health scientists have wrestled with the notion that (mainly but not exclusively nonpharmacological) interventions are highly complex and require adaptation of the experimental paradigm to permit them to be properly examined and evaluated (Craig et al., 2008; Medical Research Council, 2000; Richards & Borglin, 2011; Richards & Rahm‐Hallberg, 2015). Clear guidance is now available for researchers who wish to develop the evidence base for complex healthcare interventions, taking such interventions through a rigorous and programmatic process of intervention development, piloting, evaluation and implementation (Craig et al., 2008; Richards & Rahm‐Hallberg, 2015).

Importantly, the not yet published but forthcoming 2020 revision of the UK Medical Research Council's guidance on complex interventions research methods will stress the value of mixed methods. This involves the integration of observational research within robust experimental methods to determine not only the relative effectiveness of complex healthcare interventions but also the reasons for their effects and the views of patients and practitioners on these interventions. Taking this argument further, in a recent communication piece from a multi‐national group of mixed methods researchers concerned to maximise the benefits of integrating qualitative and quantitative data at the level of individual participants’ data in clinical trials, the authors argue that such methods “can yield insights that might be useful for understanding variation in outcomes, the mechanism by which interventions have an impact, and identifying ways of tailoring therapy to patient preference and type*”* p1 (Richards et al., 2019).

In the light of these significant advances in applied health services research methods for complex interventions, there is certainly no moral or ethical argument for abandoning the scientific method in favour of continuing untested custom and practice, practice that may in fact actively or passively harm the very people we are trying to care for. Rather, those researchers in nursing aiming to meet Kitson and colleague's challenge to *research* fundamental care (Kitson et al., 2019), should at the very least cease further observational research unconnected to intervention testing, and combine their observational insights with robust experimental methods in large scale clinical trials.

These are, therefore, exciting times for health services researchers and those of us wanting to place nursing practices on a secure evidence‐based footprint. On the horizon are new methods, new applied philosophies and new opportunities. The challenge is there for researchers in nursing, particularly those concerned to improve the scientific basis of fundamental nursing care, to take up these methods, to move from a comfortable adherence to qualitative and other observational methods and apply their expertise within multi‐disciplinary research groups involving clinical trialists, experimental methodologists and health economists.

Consequently, despite the somewhat pessimistic analysis of nursing research described in this editorial, the future indeed beckons brightly. We have models ripe for testing. We have enough observational data on important fundamental care phenomena. We have the expertise and the new methods we can use. Maybe, it is just the confidence we lack to take the scary step into experimentation. But take that step we surely must.
